# University of Pennsylvania

**Published:** 1843-03-04

**Authors:** 


UNIVERSITY OF PENNSYLVANIA.

By the Annual Catalogue just issued we find that the number of matriculants is three hundred and fifty-eight.

